# Supplementary material for: The bile acid-sensitive ion channel is gated by Ca2+-dependent conformational changes in the transmembrane domain
Source: Nat Commun. 2025 Jul 22;16:6746. doi: 10.1038/s41467-025-62038-9 (PMC12283945; doi:10.1038/s41467-025-62038-9)

Supplementary Information

The bile acid-sensitive ion channel is gated by Ca^{2+}-dependent conformational changes in the transmembrane domain

Makayla M. Freitas[1] and Eric Gouaux[1,2*]

[1]Vollum Institute, Oregon Health and Science University, 3232 SW Research Drive, Portland, OR, USA.

[2]Howard Hughes Medical Institute, Oregon Health and Science University, 3232 SW Research Drive, Portland, OR, USA.

*Correspondence to: Eric Gouaux, gouauxe@ohsu.edu

Supplementary Information

Supplementary Fig. 1: Function of hBASIC construct.

Supplementary Fig. 2: Biochemical and functional characterization of recombinant hBASIC.

Supplementary Fig. 3: Cryo-EM processing pipeline for 2 mM Ca^{2+} data set.

Supplementary Fig. 4: 2mM Ca^{2+} hBASIC cryo-EM map analysis.

Supplementary Fig. 5: Comparison of hBASIC to cASIC1a.

Supplementary Fig. 6: Cryo-EM processing workflow of EGTA data set.

Supplementary Fig. 7: EGTA, closed, hBASIC cryo-EM map analysis.

Supplementary Fig. 8. EGTA, expanded, hBASIC cryo-EM map analysis.

Supplementary Fig. 9. β-linker dynamics are coupled to transmembrane domain conformation

Supplementary Fig. 10. Intermolecular interactions between β-linkers.

Supplementary Fig. 11. Structural differences in the channel pore with and without Ca^{2+}.

Supplementary Fig. 12. Example traces and statistical analysis of IC_{50}.

Supplementary Fig. 13. Cryo-EM processing workflow of Ba^{2+}-closed data set.

Supplementary Fig. 14. Ion and surrounding density in Ca^{2+}-closed, Ba^{2+}-closed, EGTA-open, and EGTA-closed maps.

Supplementary Fig. 15. Additional current-voltage analysis and controls.

Supplementary Table 1. Site-directed mutagenesis primers

Source Data, Supplementary Fig. 2c

Source Data, Supplementary Fig. 2i

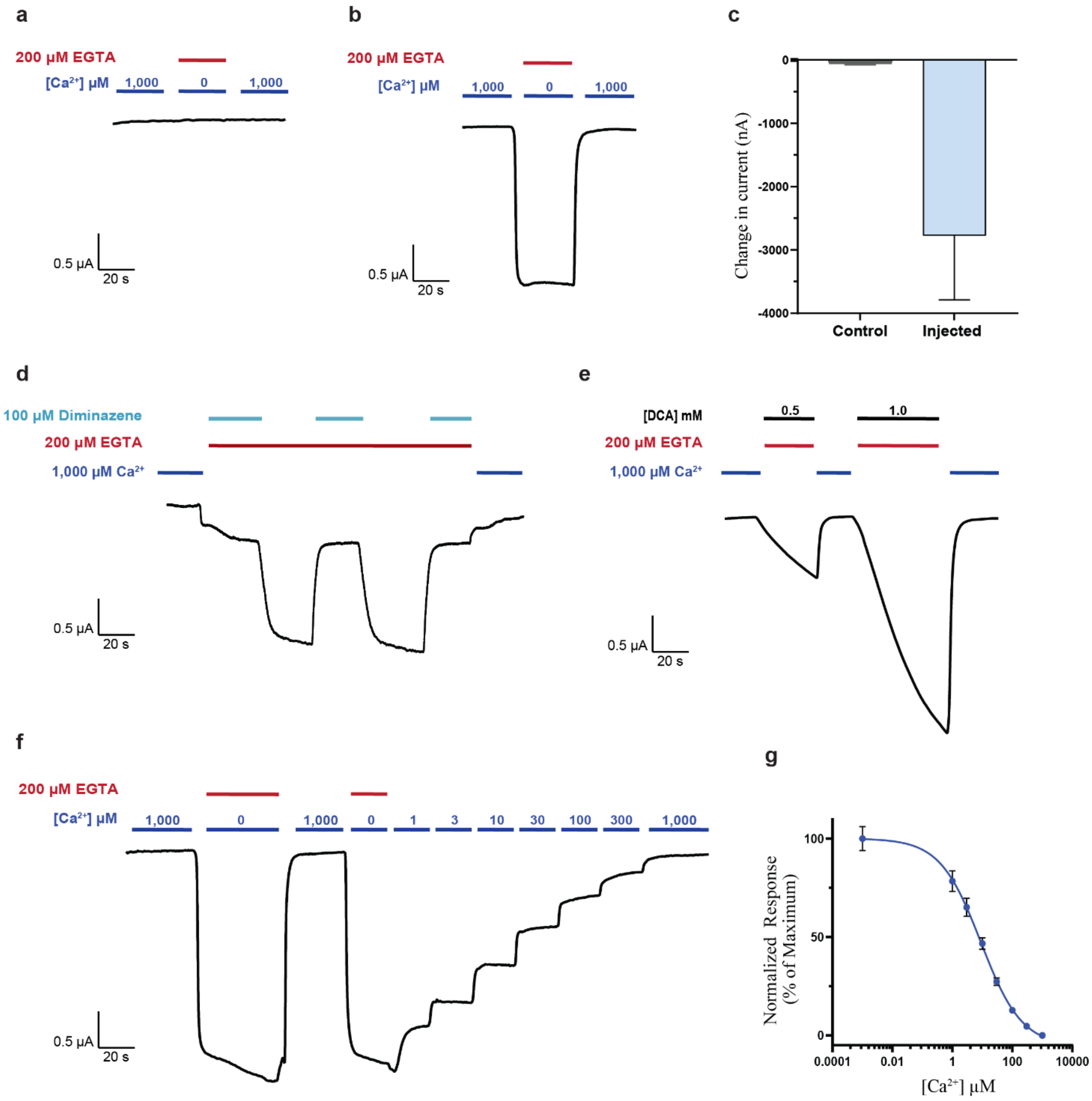

Supplementary Fig. 1. Function of hBASIC construct. (a-b) Example recording of **(a)** oocyte control and **(b)** oocyte expressing hBASIC when recorded in the presence of 1 mM Ca^{2+} or 0.2 mM EGTA. **(c)** Change in current (nA) upon Ca^{2+} removal from the bath between control oocytes and hBASIC injected oocytes. **(d)** hBASIC's inhibitor, diminazene, attenuates Ca^{2+} chelation activated currents in oocytes expressing hBASIC. **(e)** Ca^{2+} chelation activated currents can be potentiated by increasing amounts of the

bile acid, deoxycholic acid (DCA). **(f-g)** There is no significant difference in potency of Ca^{2+} inhibition of hBASIC by Ca^{2+} when perfusion of [Ca^{2+}] is performed in a 'reverse order' (i.e. increasing [Ca^{2+}], instead of decreasing [Ca^{2+}]). **(f)** A representative trace of the control experiment with the corresponding statistics analysis **(g)** that show the IC_{50} is 8μM ± 2 (n=3). Source data are provided as a Source Data file.

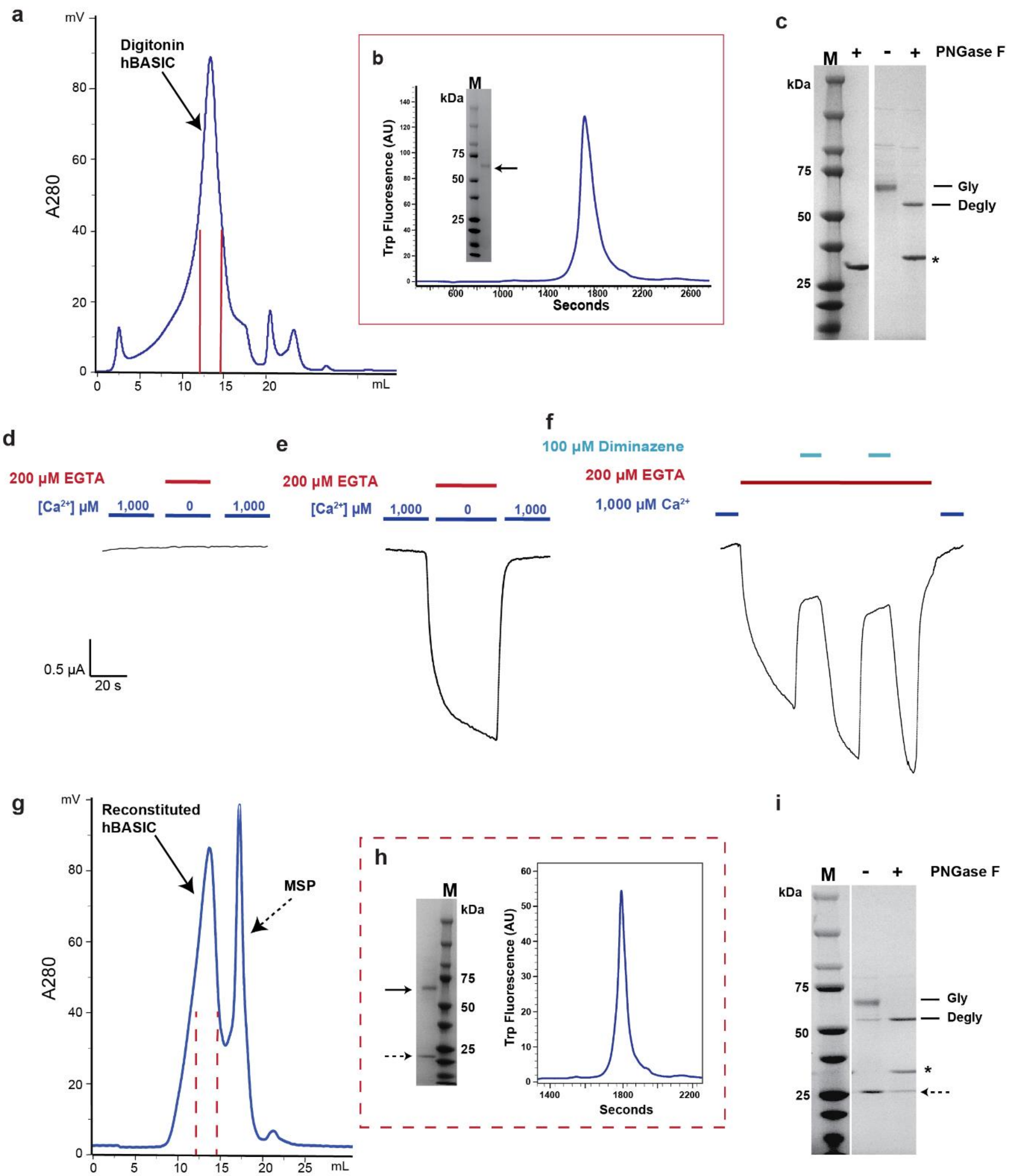

Supplementary Fig. 2. Biochemical and functional characterization of recombinant hBASIC. (a) Size exclusion chromatography (SEC) chromatogram of digitonin extracted hBASIC expressed in HEK293 cells. Solid red lines indicate peak fraction corresponding to hBASIC. **(b)** Fluorescent size exclusion

chromatography (FSEC) and SDS-PAGE gel analysis of digitonin hBASIC sample from peak fraction of SEC elution. **(c)** SDS-PAGE analysis of digitonin solubilized hBASIC with and without PNGase F treatment. Lines indicate glycosylated and deglycosylated hBASIC. Star indicates PNGase F. **(d-f)** Representative TEVC current trace recordings of **(d)** uninjected oocytes and **(e-f)** oocytes injected with digitonin purified hBASIC reconstituted into liposomes. Injected oocytes demonstrate inward sodium currents in response to the removal of Ca^{2+} via EGTA chelation. **(f)** The EGTA chelated currents are attenuated with hBASIC pore blocker, diminazene. **(g)** Example SEC chromatogram of nanodisc reconstituted hBASIC. Solid arrow represents nanodisc embedded hBASIC, while a dashed arrow represents membrane scaffold protein (MSP). Elution fraction indicated with dashed red lines. **(h)** SDS-page and FSEC analysis of nanodisc embedded hBASIC from SEC elution. **(i)** SDS-PAGE analysis of nanodisc-embedded hBASIC with and without PNGase F treatment. Lines indicate glycosylated and deglycosylated hBASIC. Star indicates PNGase F. Dashed arrow indicates MSP protein. Source data are provided as a Source Data file.

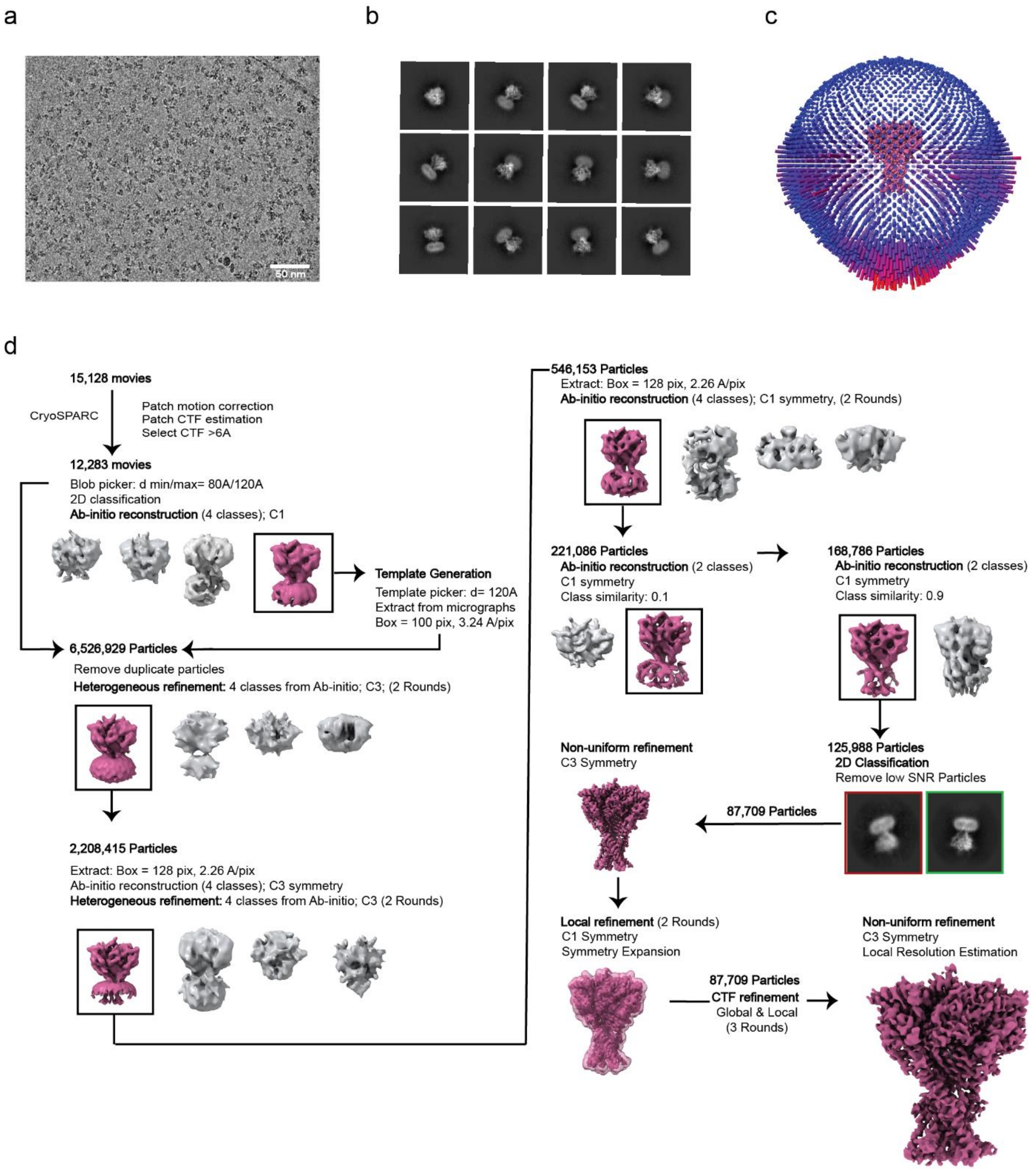

Supplementary Fig. 3. Cryo-EM processing pipeline for 2 mM Ca^{2+} data set. (a) Representative micrograph from data set. **(b)** Selected 2D classes and **(c)** angular distribution of particles included in the final map. **(d)** Data processing workflow.

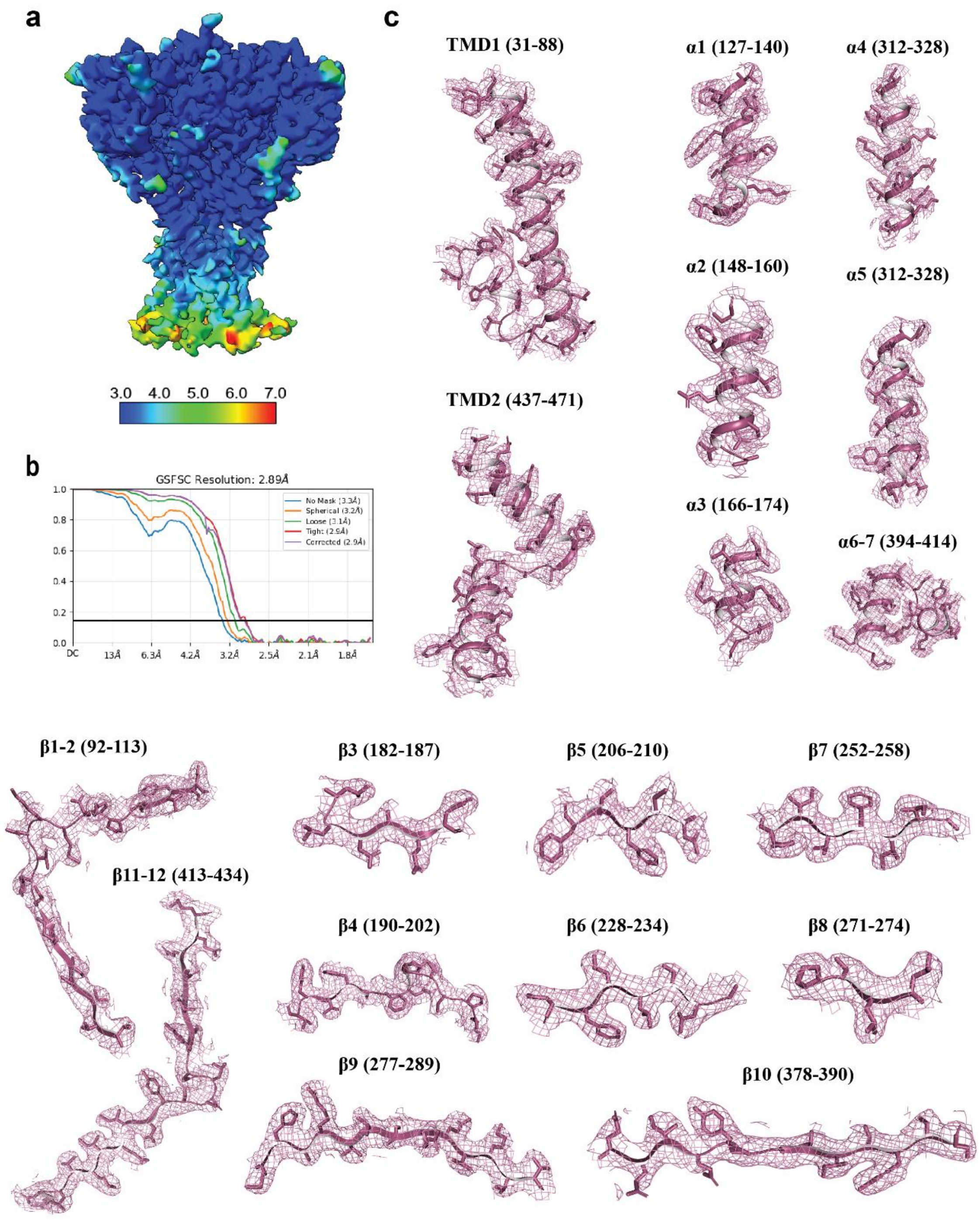

Supplementary Fig. 4. 2mM Ca^{2+} hBASIC cryo-EM map analysis. (a) Local resolution and **(b)** Fourier shell correlation (FSC) plot of 2mM Ca^{2+} hBASIC map. **(c)** Density associated with TMDs, α-helices, and β-sheets. Isomesh map features are contoured at 6.0 σ and within 2Å associated with each feature.

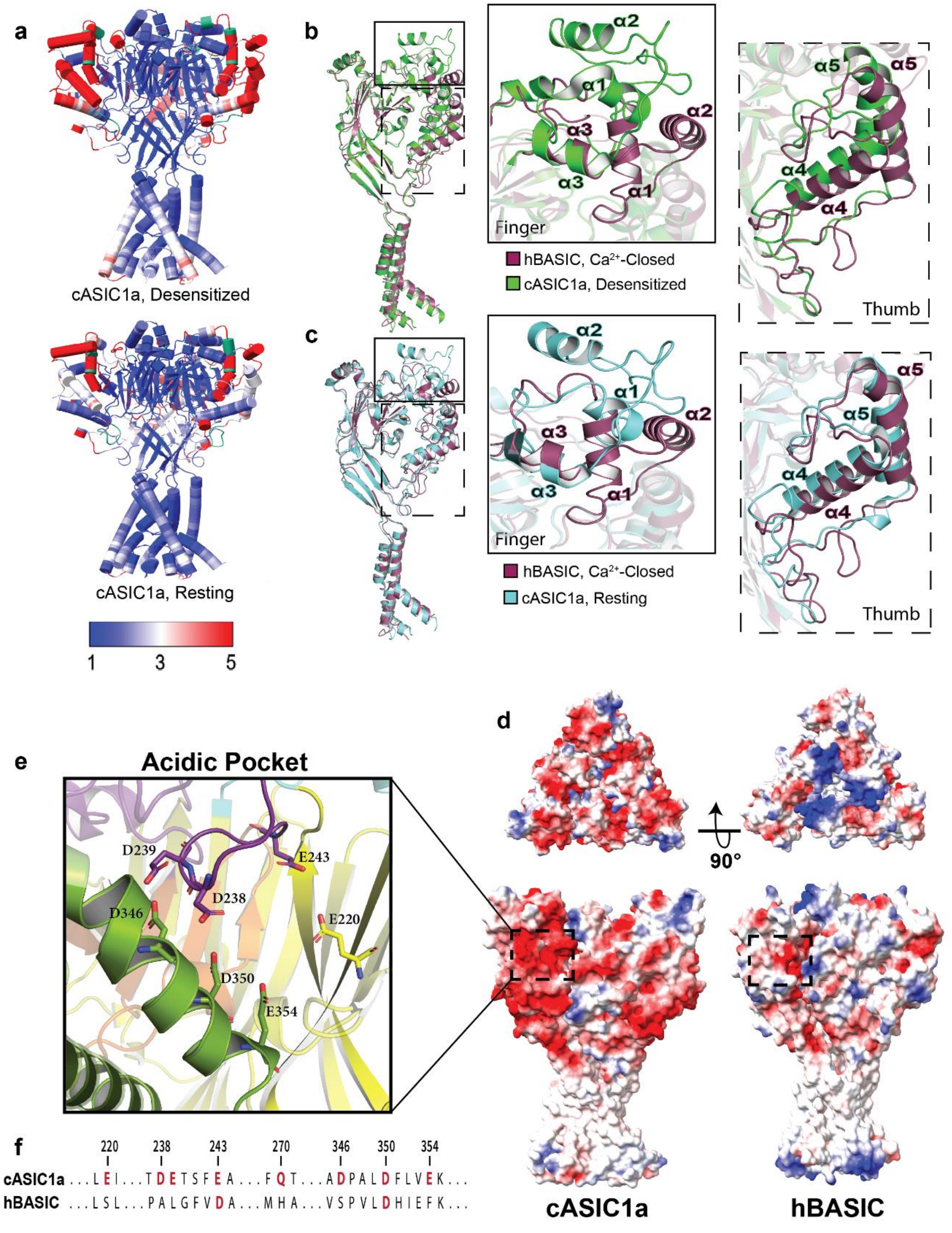

Supplementary Fig. 5. Comparison of hBASIC to cASIC1a. (a) rmsd of Ca^{2+} hBASIC in comparison to cASIC1a, desensitized [PDB:6VTK] and cASIC1a, Resting [PDB:6VTL]. Green represents unique inserts, Blue = high conservation, Red = low conservation. **(b-c)** hBASIC with Ca^{2+} structure

superimposed onto cASIC1a desensitized [PDB:6VTK] **(b)** and resting [PDB: 6VTL] **(c)**, highlighting regions of low conservation, the finger and thumb. **(d)** Models of cASIC1a and hBASIC colored by electrostatic potential, with red representing regions of negative potential through blue for positive potential. A dashed box indicates the acidic pocket of cASIC1a and the equivalent region in hBASIC. **(e)** The acidic pocket of cASIC1a, a pocket formed by intersecting regions of the finger, thumb, and palm domain. **(f)** Sequence comparison of the acidic pocket between cASIC1a and hBASIC, showing lack of sequence conservation of these acidic residues.

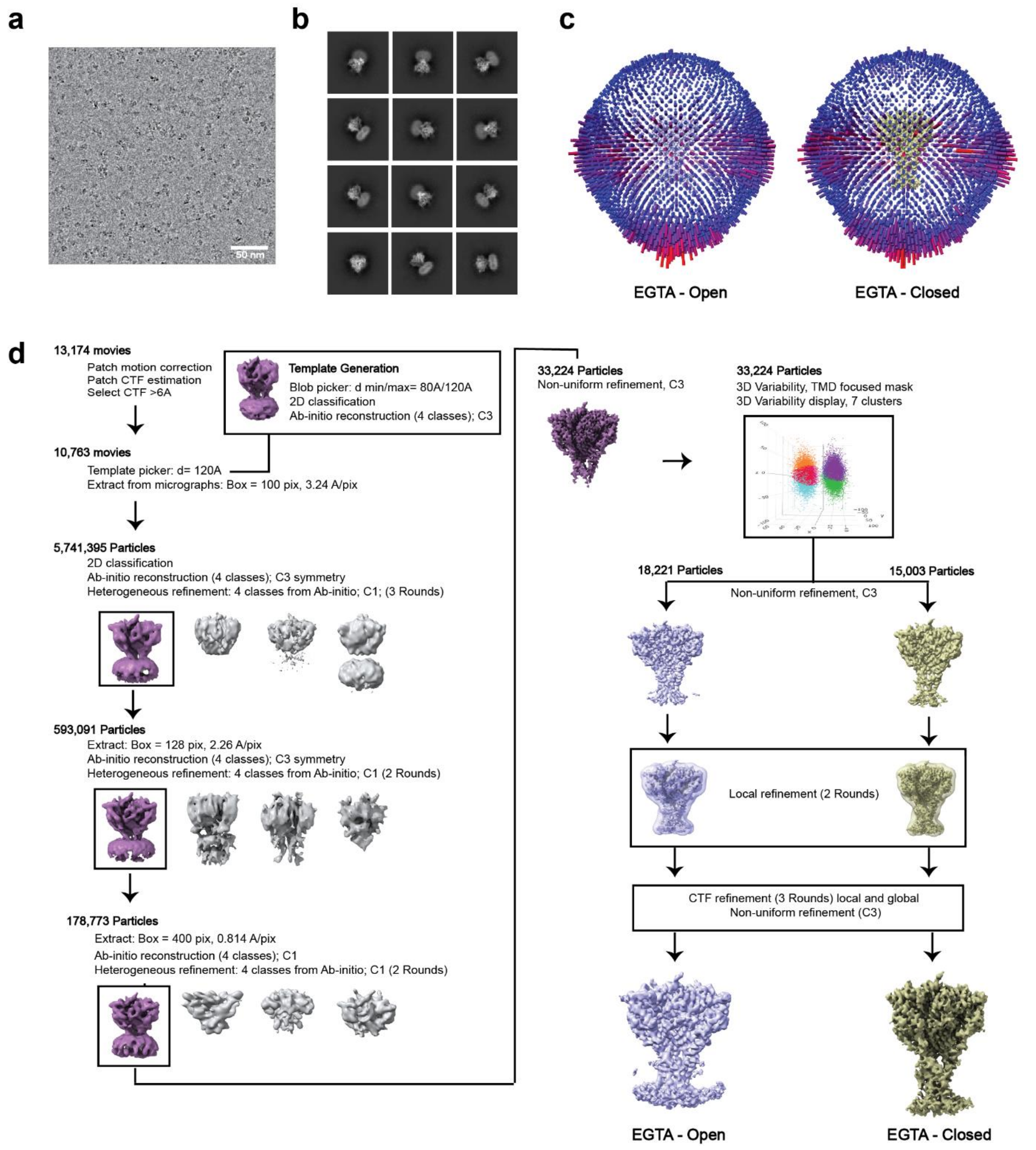

Supplementary Fig. 6. Cryo-EM processing workflow of EGTA data set. (a) Representative micrograph from data set. **(b)** Selected 2D classes and **(c)** angular distribution of particles included in the final maps. **(d)** Data processing workflow.

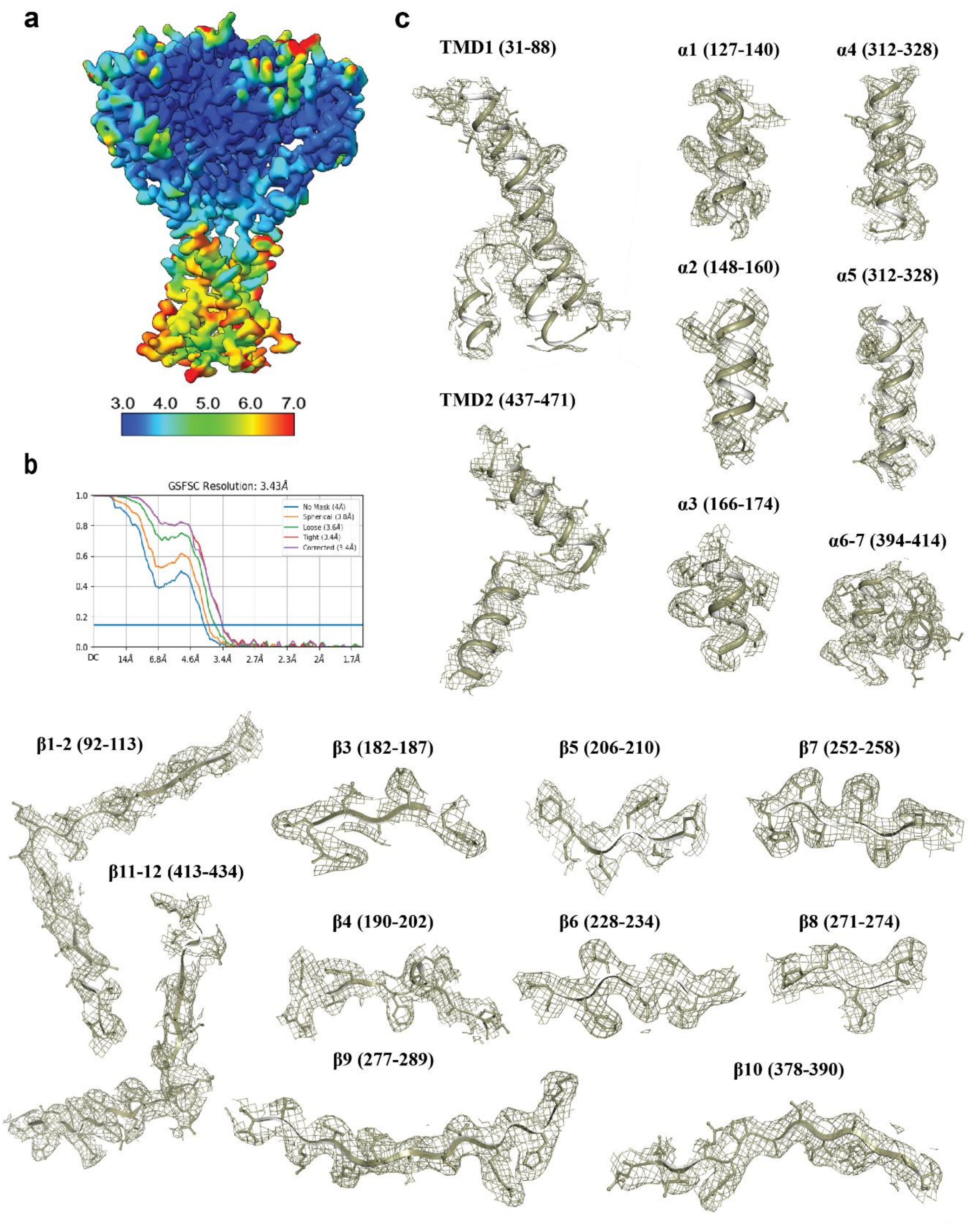

Supplementary Fig. 7. EGTA, closed, hBASIC cryo-EM map analysis. (a) Local resolution and **(b)** Fourier shell correlation (FSC) plot of EGTA, closed, hBASIC map. **(c)** Density associated with TMDs, α-

helices, and β-sheets. Isomesh map features are contoured at 5.0 σ and within 2Å associated with each feature.

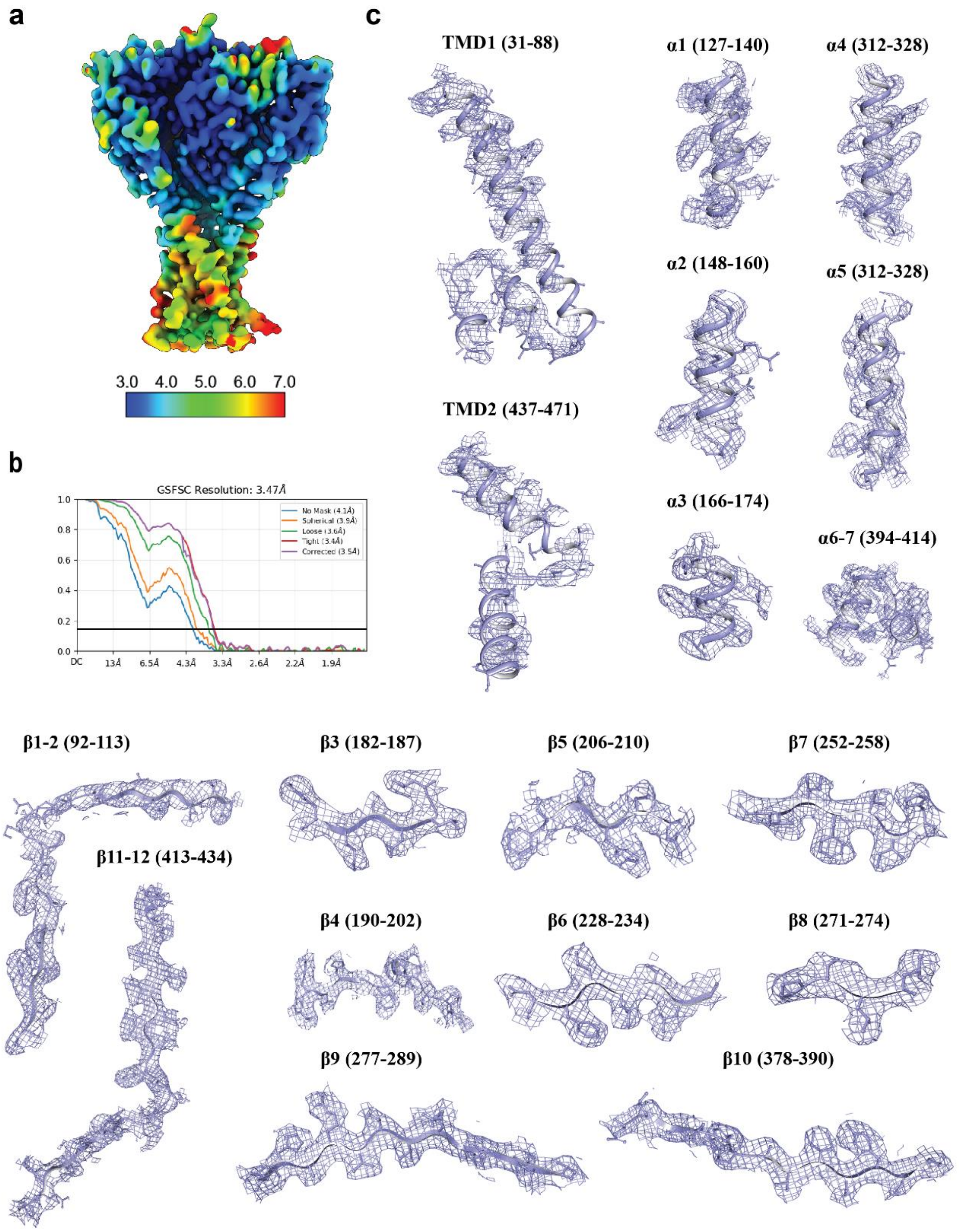

Supplementary Fig. 8. EGTA, expanded, hBASIC cryo-EM map analysis. (a) Local resolution estimation of EGTA, expanded map. **(b)** Fourier shell correlation (FSC) plot of corresponding map. **(c)** Density, contoured at 5.0 σ and within 2Å associated with TMDs, α-helices, and β-sheets.

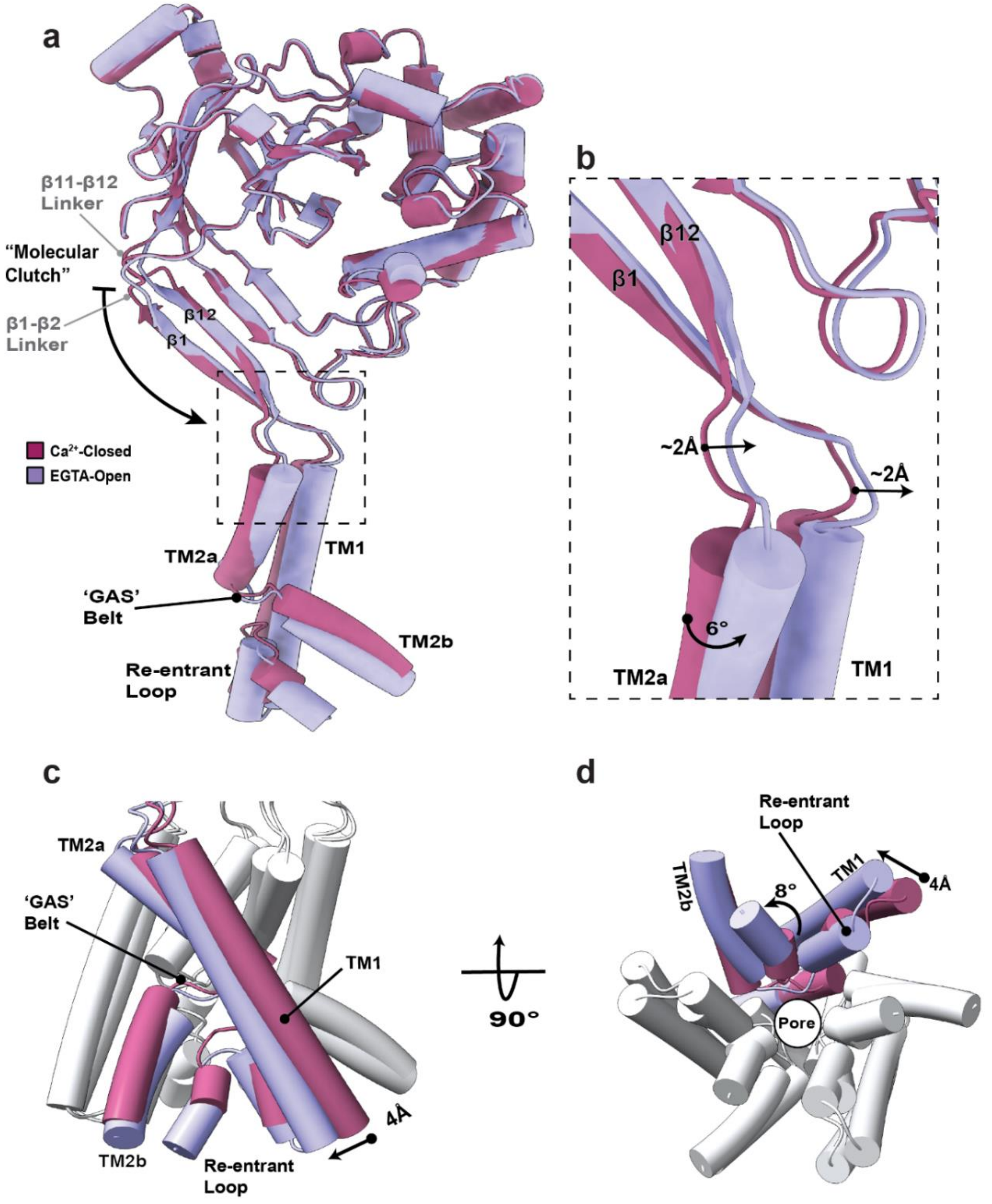

Supplementary Fig. 9. β-linker dynamics are coupled to transmembrane domain conformation. (a) Superposition of a single protomer of hBASIC in the Ca^{2+}-closed and EGTA-open states, illustrating the structural connection between the molecular clutch and the transmembrane domain (TMD). **(b)** Close-up view of the junction between the β1-β12 linkers and the TMD. **(c-d)** Ca^{2+}-dependent conformational

changes of the TMD, shown from **(c)** a side view and **(d)** a bottom view, highlighting the displacement of TM1, TM2b, and the re-entrant loop.

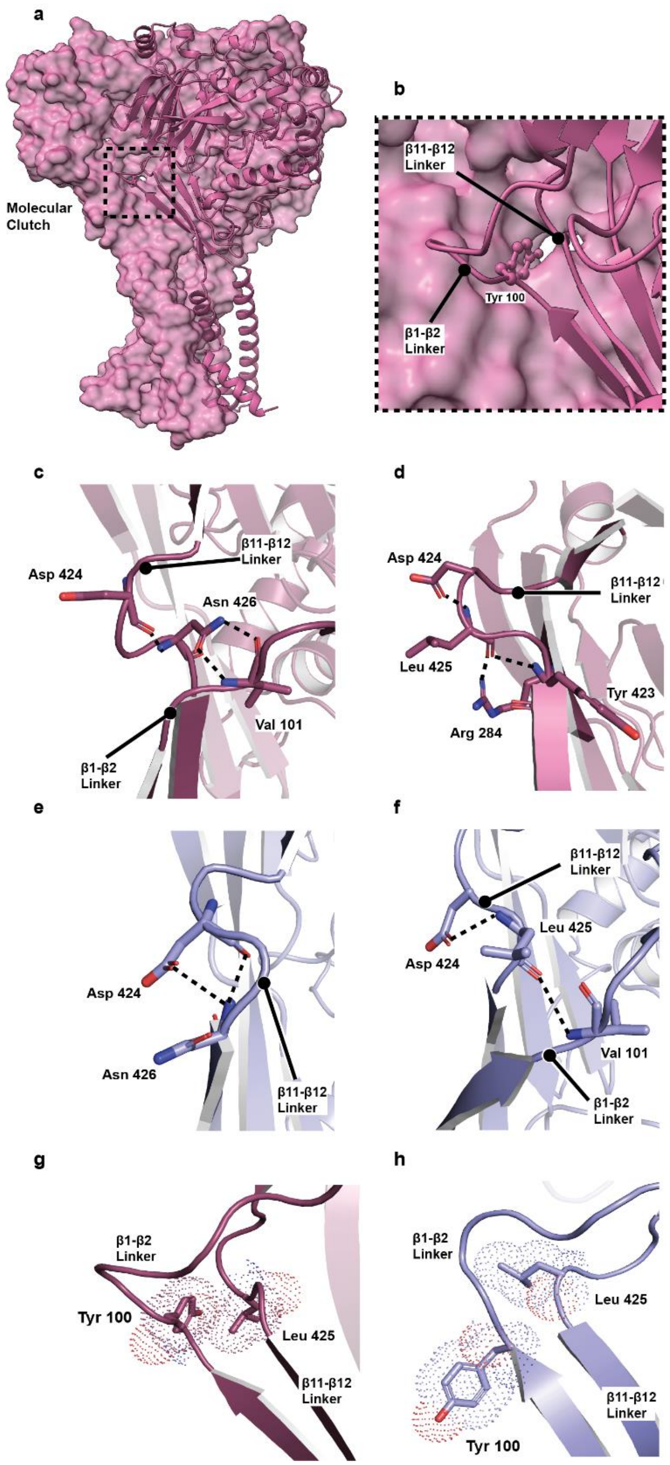

Supplementary Fig. 10. Intermolecular interactions between β-linkers. (a) The molecular clutch is highlighted on an individual subunit of the Ca^{2+}-closed model. **(b)** Close-up view of the β-linkers, emphasizing the localization of Tyr100 within the β1-β2 linker. **(c-f)** Local interactions involving Leu425

and Asn426, residues of β11-β12 linker, interacting with residues of the β1-β2 linker in the **(c-d)** Ca^{2+}-closed state and **(e-f)** EGTA-open state. **(g-h)** Ca^{2+}-dependent dynamics of Tyr100 and Leu425. **(g)** In the Ca^{2+}-closed model, Tyr100 sterically hinders the reorientation of Leu425. **(h)** In the EGTA-open model, removal of Ca^{2+} displaces Tyr100, allowing Leu425 to reorient.

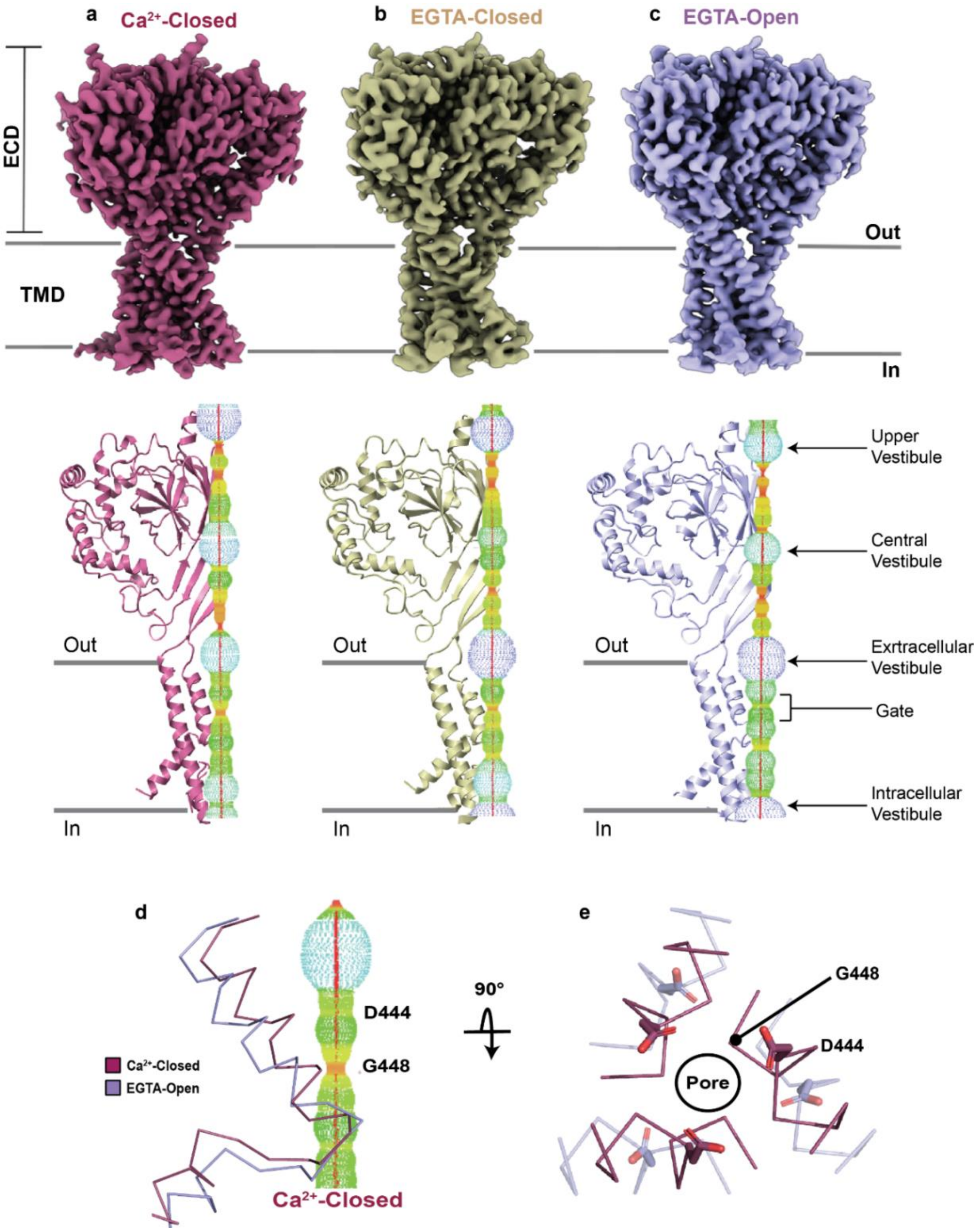

Supplementary Fig. 11. Structural differences in the channel pore with and without Ca^{2+}. (a-c) Corresponding maps, models, and pore profile calculated with HOLE software of **(a)** Ca^{2+}-closed, **(b)** EGTA-closed, and **(c)** EGTA-open state. Pore radii: red < 1.15 Å, green < 2.3 Å, < purple. **(d)** TM2a of hBASIC model Ca^{2+}-closed superimposed onto EGTA-open state, with corresponding pore profile of Ca^{2+}-

closed calculated with HOLE software. **(e)** Top-down view of TM2a hBASIC models, highlighting residues G448 and D444.

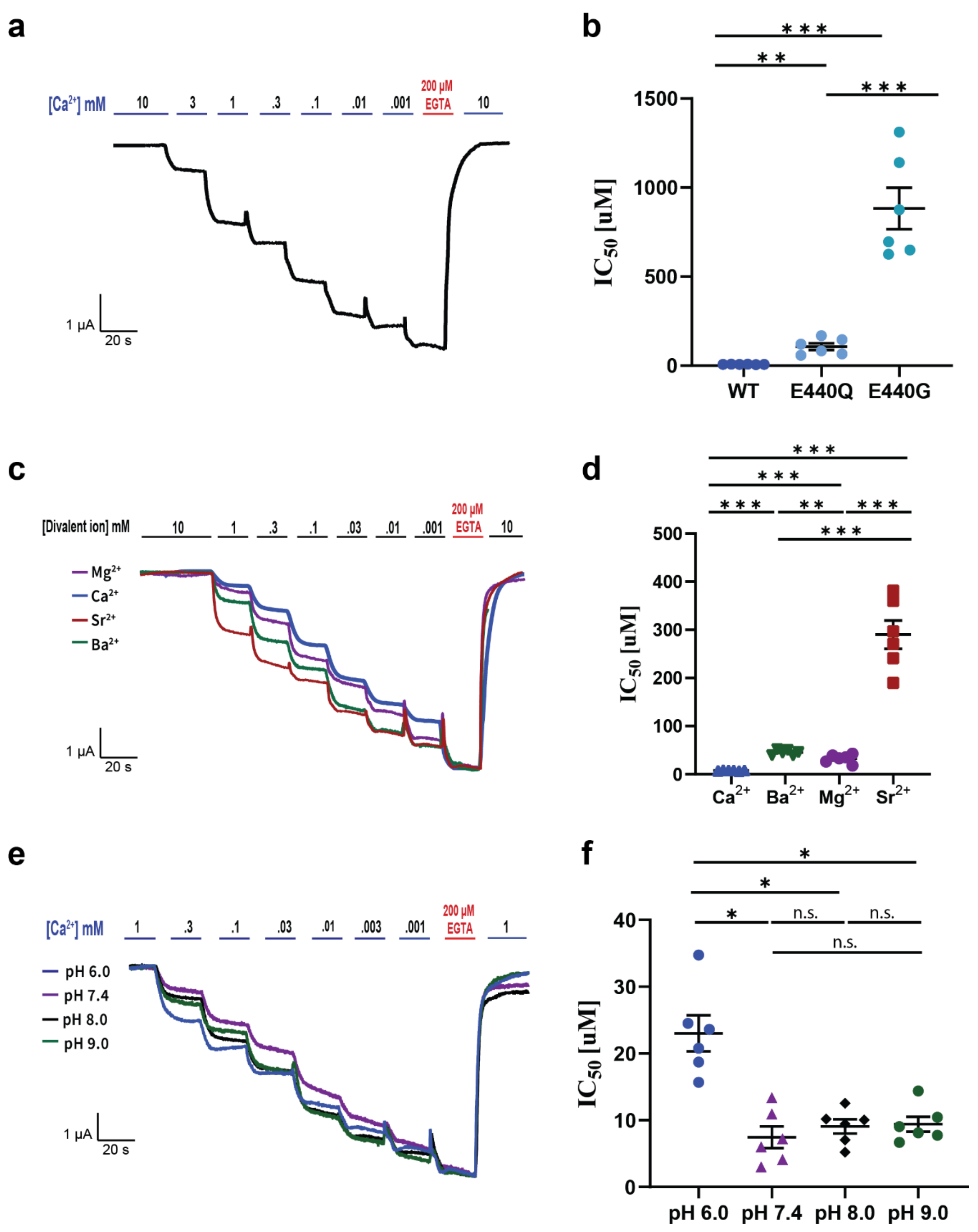

Supplementary Fig. 12. Example traces and statistical analysis of IC_{50}. (a) Example recording of an inhibition dose-response curve of Ca^{2+} of an oocyte injected with mutant E440Q. **(b)** Statistical analysis of IC_{50} of WT, and mutants, E440Q and E440G (n=6). WT versus E440Q, P= 1.4 E-4; WT versus E440G, P=9.9 E-6, E440Q versus E440G, P=3.1E-5. **(c)** Example traces of dose-response recordings of oocytes expressing hBASIC for inhibition by Mg^{2+}, Ca^{2+}, Sr^{2+}, and Br^{2+}. **(d)** Statistical analysis of IC_{50} of inhibition

by divalent ions (n=6). Ca^{2+} versus Ba^{2+}, P=1.1 E-7; Ca^{2+} versus Mg^{2+}, P=2.7 E-5, Ca^{2+} versus Sr^{2+}, P=1.2 E-6, Ba^{2+} versus Mg^{2+}, P=4.7 E-4, Ba^{2+} versus Sr^{2+}, P=7.3 E-6, Mg^{2+} versus Sr^{2+}, P=2.9 E-6. **(e)** Example traces of dose-response recordings of oocytes expressing hBASIC for inhibition by Ca^{2+} at pH 6.0. **(f)** Statistical analysis of IC_{50} of inhibition by Ca^{2+} at different pH values (n=6). pH6 versus pH7.4, P= 0.019; pH6 versus pH8, P=0.026, pH6 versus pH9, P=0.028, pH7.4 versus pH8, P=0.127, pH7.4 versus pH9, P=0.103, pH8 versus pH9, P=0.42. **(b, d, f)** Statistical significances are shown as $*P < 0.05$, $**P < 0.01$, $***P < 0.001$. Significances were evaluated using two-tailed Student's *t*-tests. Source data are provided as a Source Data file.

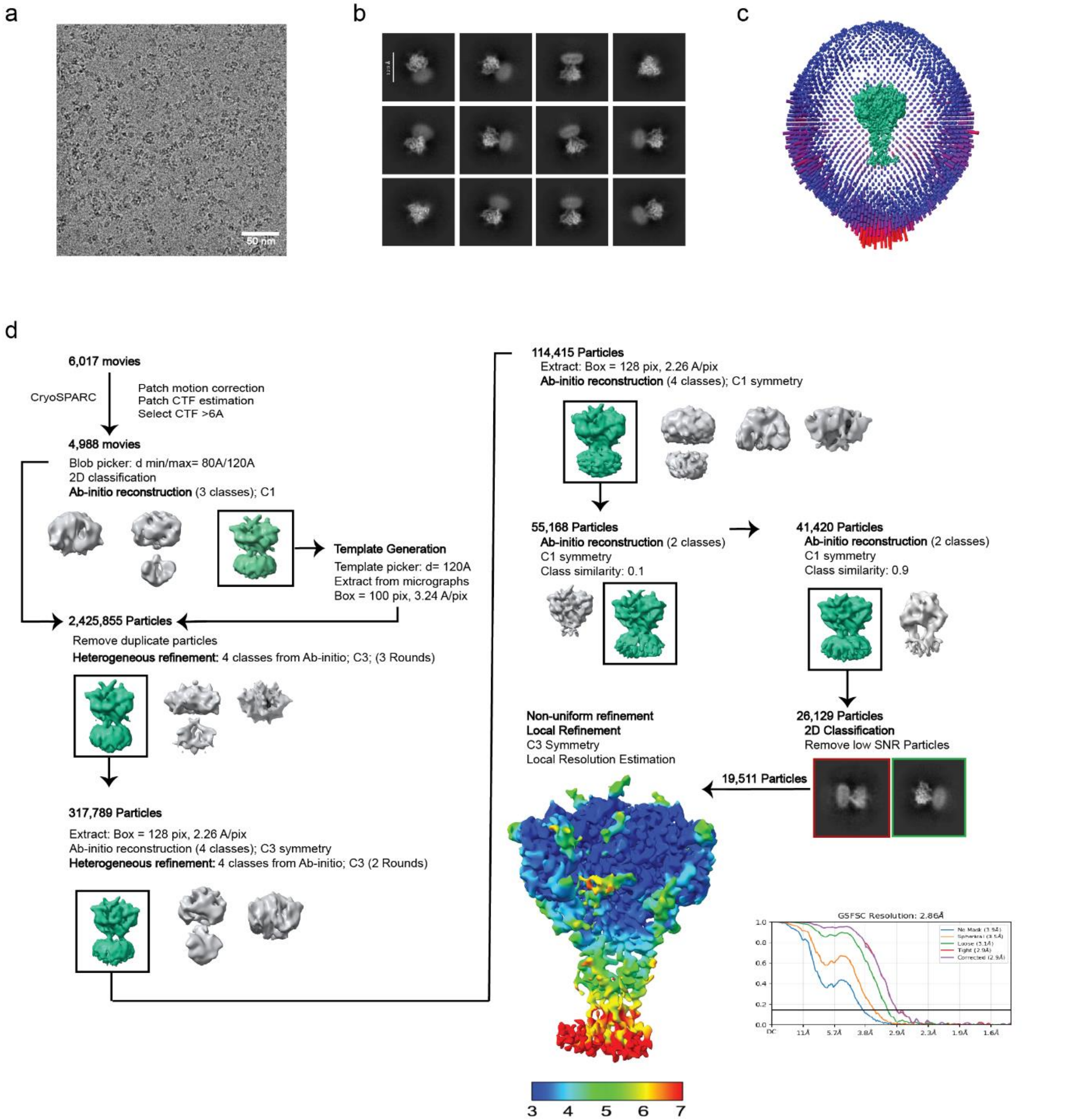

Supplementary Fig. 13. Cryo-EM processing workflow of Ba^{2+}-closed data set. (a) Example micrograph from the dataset. **(b)** Representative 2D class averages and **(c)** particle orientation distribution used for final reconstructions. **(d)** Overview of the data processing pipeline.

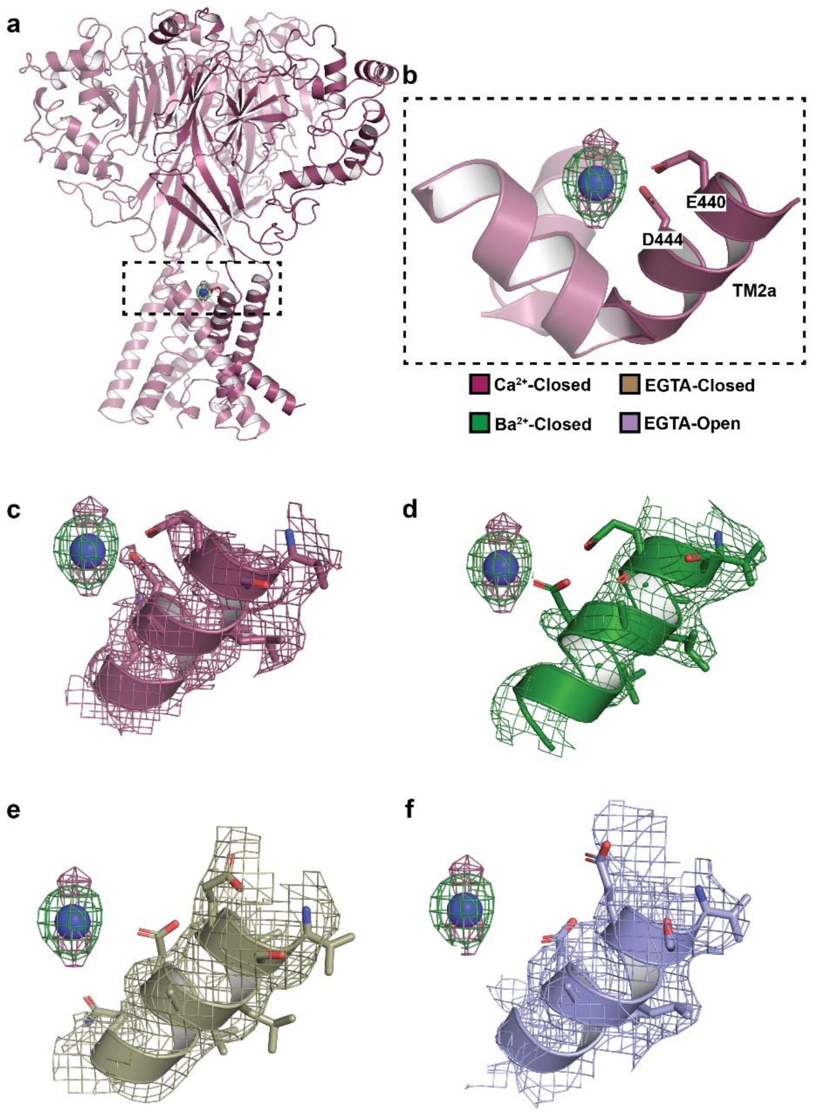

Supplementary Fig. 14. Ion and surrounding density in Ca^{2+}-closed, Ba^{2+}-closed, EGTA-open, and EGTA-closed maps. (a) Ca^{2+}-closed model highlighting the localization of the ion-like density at the top of the pore. **(b)** Close-up view of TM2a Ca^{2+}-closed model with the ion-like density in the Ca^{2+}-closed and Ba^{2+}-closed maps, with the absence of a corresponding density in the EGTA-open and EGTA-closed maps. **(c-f)** Local ion density and surrounding residue density at the top of the pore, displayed at the same contour level for the **(c)** Ca^{2+}-closed, **(d)** Ba^{2+}-closed, **(e)** EGTA-closed, and **(f)** EGTA-open maps. In panels **c-f**, in instances where side chain density is weak, side chain positions were first defined by density features for

Cβ and subsequently by utilizing the preferred protomers. Density is contoured at 5.0σ and within 2 Å of the transmembrane domains (TMDs).

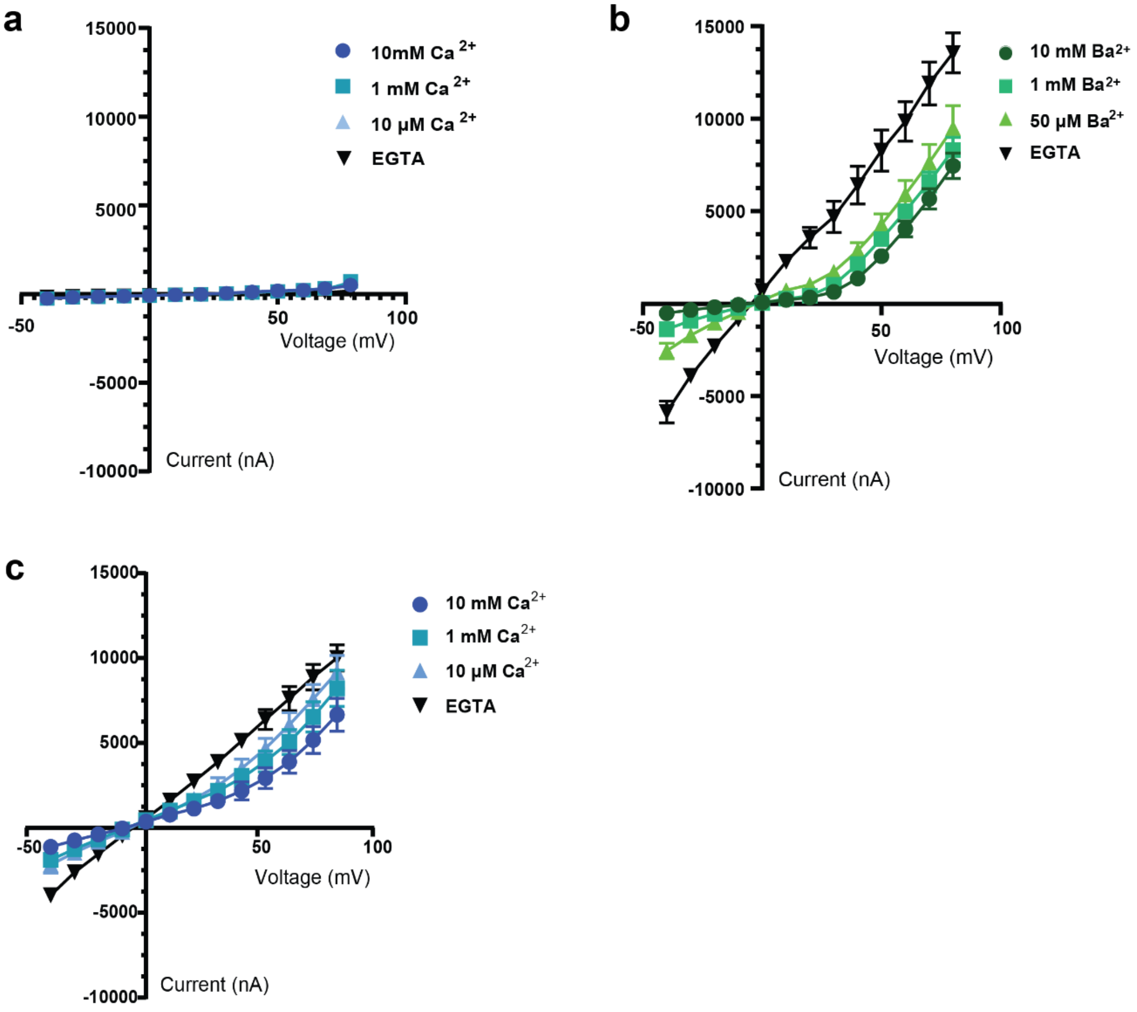

Supplementary Fig. 15. Additional current-voltage analysis and controls. (a) Current-voltage (IV) relationship of uninjected oocytes recorded (n=3) in the presence of [$Ca^{2+}{}_{o}$]. **(b)** IV plot of oocytes (n=7) injected with wild-type hBASIC RNA, measured in the presence of varying Ba^{2+} concentrations. **(c)** IV curves of oocytes (n=7) injected with mutant hBASIC RNA, E440Q, recorded under different [$Ca^{2+}{}_{o}$]. Source data are provided as a Source Data file.

Supplementary Table 1. Site-directed mutagenesis primers

Mutant	Primer Sequence
E440G_Forward	ggctgtgagcgtatcagggctgctggctgacctgggaggacagcttg
E440G_Reverse	gtcctcccaggtcagccagcagccctgatacgctcacagccttctgctgctggg
E440Q_Forward	gctgtgagcgtatcacagctgctggctg
E440Q_Reverse	cagccagcagctgtgatacgctcacagc

Source Data, Supplementary Fig. 2c.

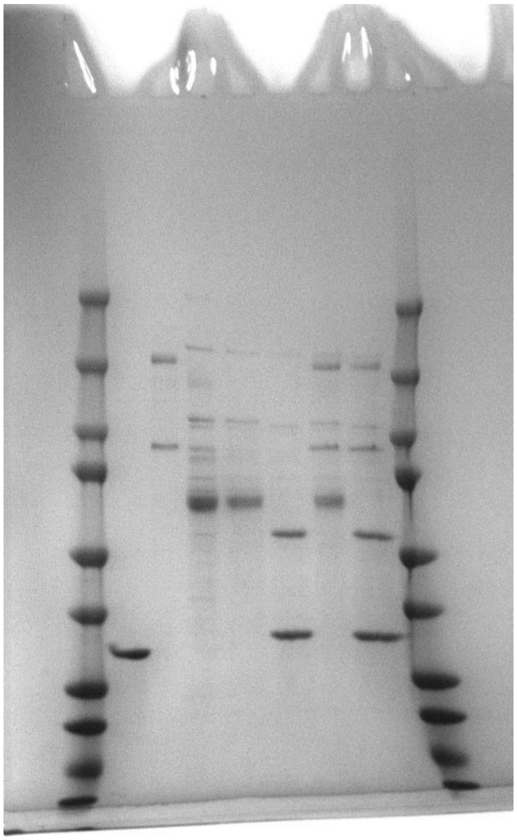

Source Data, Supplementary Fig. 2i.

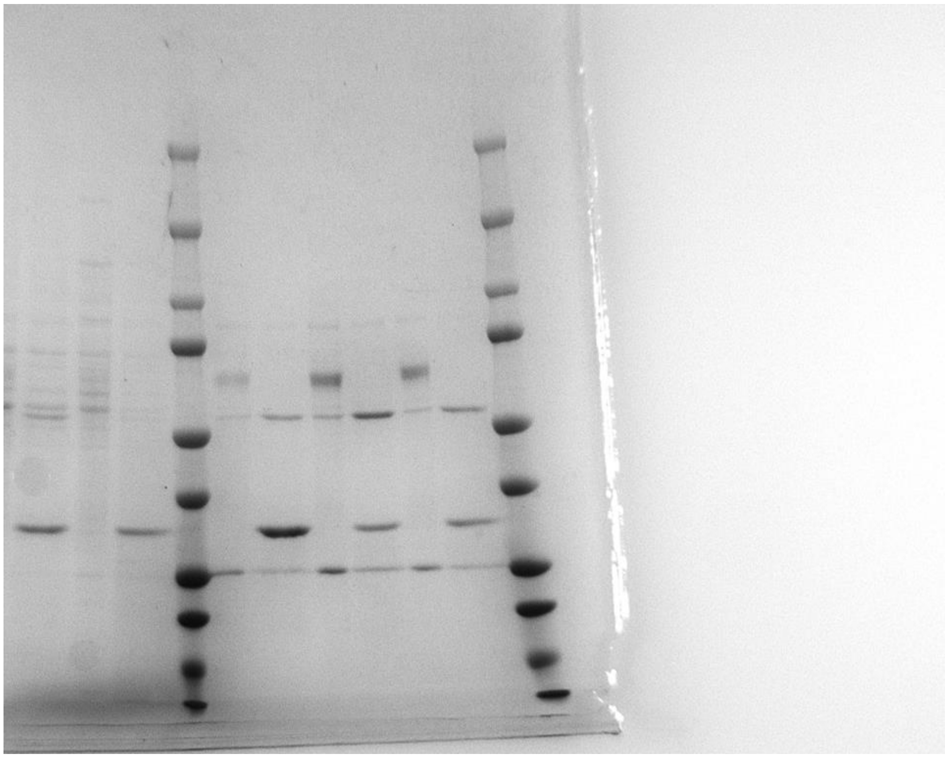

Supplement: Supplementary file 1 — Supplementary Information [file 41467_2025_62038_MOESM1_ESM.pdf]
